# A novel transformer using dynamic range-enhanced discrete cosine transform for detecting bean leaf diseases

**DOI:** 10.3389/fpls.2025.1624373

**Published:** 2025-08-29

**Authors:** Ibrahim Furkan Ince, Harisu Abdullahi Shehu, Shaira Osmani, Faruk Bulut

**Affiliations:** ^1^ Department of Software Engineering, Istinye University, Istanbul, Türkiye; ^2^ School of Engineering and Computer Science, Victoria University of Wellington, Wellington, New Zealand; ^3^ Women in Tech, Washington DC, United States; ^4^ School of Computer Science and Electronic Engineering, University of Essex, Colchester, United Kingdom; ^5^ Software Engineering, Istanbul Aydın University, Istanbul, Türkiye

**Keywords:** bean leaf diseases classification, transformer deep learning model, image 31 preprocessing, image classification, agricultural informatics, frequency weighting. 32 33

## Abstract

**Introduction:**

Early detection of diseases on bean leaves is essential for preventing declines in agricultural productivity and mitigating broader agricultural challenges. However, some bean leaf diseases are difficult to detect even with the human eye, posing significant challenges for machine learning methods that rely on precise feature extraction.

**Methods:**

We propose a novel approach, DCT-Transformers, which combines a preprocessing technique, dynamic range enhanced discrete cosine transform (DRE-DCT) with Transformer-based models. The DRE-DCT method enhances the dynamic range of input images by extracting high-frequency components and subtle details that are typically imperceptible while preserving overall image quality. Transformer models were then used to classify bean leaf images before and after applying this preprocessing step.

**Results:**

Experimental evaluations demonstrate that the proposed DCT-Transformers method achieved a classification accuracy of 99.56% (precision: 0.9916, recall: 0.9912, F1-score: 0.9912) when using preprocessed images, compared to 95.92% when using non-preprocessed images. Moreover, the method outperformed state-of-the-art approaches (all below 94%) and similar studies (all below 98.5%).

**Discussion:**

These findings indicate that enhancing feature extraction through DRE-DCT significantly improves disease classification performance. The proposed method offers an efficient solution for early disease detection in agriculture, contributing to improved disease management strategies and supporting food security initiatives.

## 1 Introduction

Bean diseases represent a significant challenge to global agriculture, causing economic losses estimated at billions of dollars annually. These diseases not only threaten agricultural productivity, but also seriously affect food security on a global scale. Agriculture remains the backbone of economies worldwide, providing livelihoods and economic income to billions, with bean crops being a staple food in many countries for their nutritional and economic value (Jones, 2021). Effective management and early detection of bean foliar diseases are key to maintaining crop health and ensuring optimal agricultural productivity (Myers and Kmiecik, 2017). Diseases that damage bean leaves can significantly reduce yields, affecting both the quality and quantity of production (Tavakoli et al., 2021; Singh et al., 2023). Early recognition and preventive treatment of these diseases allow farmers to mitigate their spread, reduce dependence on costly and environmentally damaging chemical treatments, and maintain long-term agricultural practices.

Traditional disease detection methods are primarily based on manual inspection, which requires a lot of work, takes a long time, and is prone to human mistake (Shoaib et al., 2023). In this era of technological advancement, there is a growing need for accurate and efficient automated detection systems that can enhance early disease detection, facilitate early intervention, and control disease outbreaks. The integration of artificial intelligence and agriculture has recently led to changes, especially in the use of plant disease detection (Shaikh et al., 2022; Uryasheva et al., 2022). Deep learning (DL) and computer vision have greatly increased the accuracy of disease detection in various crops using large datasets to increase diagnostic accuracy (Abbas et al., 2021; Bhatt et al., 2021). As DL models become increasingly sophisticated, they offer innovative solutions that go beyond traditional methods, providing farmers and agricultural professionals with timely and accurate information to support better decision-making. Breakthroughs in hardware technologies have empowered these applications and enabled complex computations to be performed efficiently (Marco-Detchart et al., 2023). These technological advances highlight the potential of DL beyond agriculture, showing impressive results in tasks such as crop classification, yield prediction, and disease detection using convolutional neural networks (CNNs) (Singh et al., 2023; Irmak and Saygılı, 2024; Ni, 2024; Pandey et al., 2024).

Incorporating Discrete Cosine Transform (DCT) (Ragaglia et al., 2024) into deep learning frameworks has been heralded as a pioneering approach to increase the accuracy and reliability of disease detection in crops such as beans. DCT helps transform images into the frequency domain, allowing deep learning models to more effectively distinguish complex patterns in data, thereby facilitating more accurate disease detection (Wang et al., 2020). A pioneering effort by (Kamble et al., 2023) illustrates the versatility of DCT using deep learning, creating hybrid models that improve traditional applications such as biometrics and extending them to agricultural practices, enhancing the feature extraction process. In addition, DCT is important for improving highly dynamic image processing in environments where conditions change, while maintaining the consistency and reliability of detection processes. Additionally, advances such as the FormerLeaf model by (Thai et al., 2023), which uses vision transformers, shows better performance when integrated with DCT methodologies. Such combinations provide better adaptability for multiple crops, not just cassava, and offer a template that can be extended to beans and similar crops.

A comparative analysis of different CNN architectures applied to this dataset highlights the continued development and optimization of DL approaches for improving agricultural practices. Additionally, A Frontiers team in Plant Science investigated “Plant Leaf image retrieval systems based on deep metric learning” as another approach for detecting plant leaf disease. Their team investigated how merging deep learning methods with object detection improves plant leaf disease detection accuracy using feature extraction and retrieval systems (Peng and Wang, 2022).

This study presents a new approach for accurate bean leaf disease detection using a Transformer Deep Learning model. Using a novel DRE-DCT (Dynamic Range Enhanced Discrete Cosine Transform) method to pre-process input images in both training and test datasets, we achieve 99.56% classification accuracy. This modified DCT technique incorporates frequency weighting in both the direct and inverse equations. The DRE-DCT process is applied to blocks of input images using stepwise transformations, increasing the dynamic range, extracting details and high-frequency features that are often not visible to the naked eye, while preserving the original image quality. This improvement is facilitated by the DCT’s excellent compression capability. This method effectively captures the complex features of diseased leaves, resulting in perfect classification accuracy when the preprocessed images are analyzed using the Transformer Deep Learning model. Furthermore, we observe that the classification accuracy improves when the DCT block size is closely matched to the transformer patch size 16×16, achieving maximum performance (100% accuracy on a single fold) with block sizes of 14, 15, and 16. Experimental results highlight the effectiveness of this approach, which shows significant potential for advances in disease treatment and control in agricultural practices. Our tests on the iBean leaf disease dataset provide up to 100% accuracy (on a single fold) with DCT block sizes of 14, 15, and 16 when using preprocessed images with the Transformer model. Furthermore, we evaluate different deep learning models with and without a preprocessing step, confirming the improvements attributed to the DRE-DCT method.

Our research will benefit from these advanced techniques, especially through the integration of DCT with transfer learning to enhance the accuracy of disease prediction by improving image quality and feature extraction, as highlighted in various studies. The contributions of this work are as follows:

1. Proposed a transfer learning-based approach using transformers for the detection of bean leaf diseases. As transfer learning leverages knowledge from pre-trained models on a different domain, it is anticipated that this approach will improve detection accuracy by adapting already learned features to the specific task of disease detection.
2. Introduced a DCT method for enhancing the quality of images. The DCT method aims to improve image representation by highlighting frequency-based features. As such, it is anticipated that the method will work well in predicting bean leaf diseases when combined with transformer models, since these models benefit from enhanced input data that accentuates key patterns, reducing noise and improving feature extraction.
3. Demonstrates the generalizability of the method proposed, achieving an accuracy of 99.56%, outperforming state-of-the-art deep learning methods and similar studies that reported lower accuracies.

The paper is structured as follows: in part 2, the materials and methods used are discussed; Section 3 shares the experimental results; and Section 4 presents conclusions and suggestions for future work.

## 2 Proposed method

**Figure 1:**
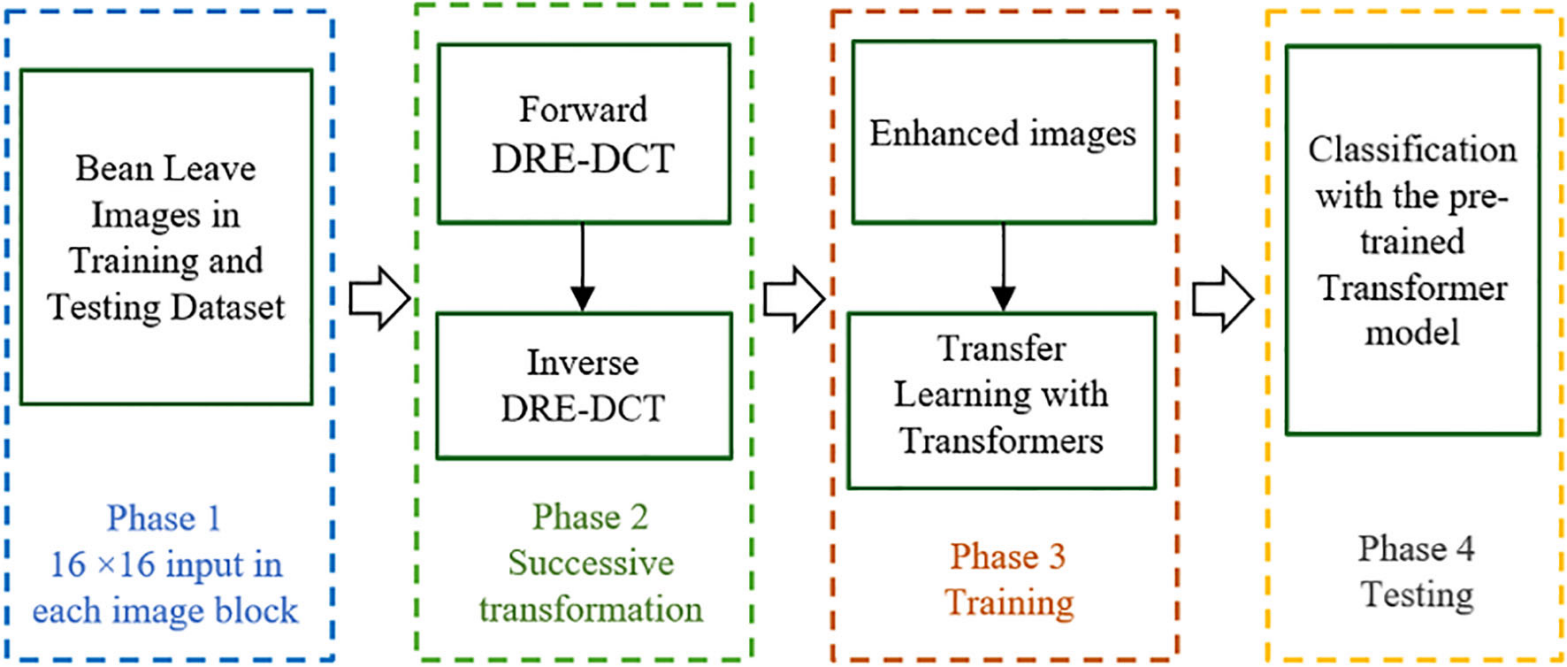
The flowchart of the proposed method.

**Figure 1** Illustrates the general structure of the proposed method, presented in four phases in the flowchart.

The proposed image enhancement algorithm is designed to improve visual quality by leveraging localized frequency transformations through the Dynamic Range Enhanced Discrete Cosine Transform (DRE-DCT). Firstly, the input image is subdivided into blocks and within each block, a horizontal DRE-DCT is applied to transform spatial information into frequency components across rows, which is followed by a vertical DRE-DCT applied column-wise to accomplish a comprehensive 2D (Two Dimensional) frequency transformation for each RGB channel. The algorithm then employs an inverse DCT process, beginning with columns and then proceeding with rows, to revert the data to its spatial domain. This successive forward and inverse DRE-DCT operation increases the dynamic range of the pixels in the blocks, where the high frequency components are revealed and extracted. By integrating the enhanced images using transfer learning with Transformer models, the algorithm facilitates superior image analysis and recognition capabilities in machine learning applications.

### 2.1 Dynamic range enhanced discrete cosine transform

An image or signal can be converted from the spatial domain to frequency domain using a mathematical method known as DCT. It reflects the data as a sum of cosine functions fluctuating at different frequencies. Simply put, it breaks down an image or signal into individual parts and highlights patterns that differ in both intensity and frequency. DCT is generally used in image compressions, like as JPEG, because it divides images into parts of different importance, allowing for efficient data reduction. By focusing on the most important frequency components, DCT can compress images while preserving most of the visual information (Ince et al., 2022).

The Discrete Cosine Transform (DCT) (Ragaglia et al., 2024) is an orthogonal transform method that, in its ideal unmodified form, can losslessly reconstruct the input DCT coefficients when processed without modification (except for numerical accuracy issues). The ability of DCT to compress significant energy into a small number of coefficients can be used to effectively represent the fundamental characteristics of the input signal. By focusing on these key factors and suppressing noise and minor components, the relative differences in salient features become more apparent. DCT is an orthonormal transform, where 1D forward DCT and 1D inverse DCT are defined by Equations 1, 2, respectively. Here, the special condition in Equation 3 is applied as follows:


(1) $$\begin{array}{c} y\left(k\right)=\sqrt{\frac{2}{N}}\alpha \left(k\right)\sum_{n=0}^{N-1}x\left(n\right)\cos\frac{\left(2n+1\right)k\pi }{2N}\\ \;k=0,\;1,\ldots N-1 \end{array}$$

(2) $$\begin{array}{c} x\left(n\right)=\sqrt{\frac{2}{N}}\sum_{k=0}^{N-1}\alpha \left(k\right)y\left(k\right)\cos\frac{\left(2n+1\right)k\pi }{2N}\\ \;n=0,\;1,\ldots N-1 \end{array}$$

(3) $$\alpha (0)=\frac{1}{\sqrt{2}};\;\alpha (k)=1;\;k\neq 0.$$

After converting the signal using DCT, different frequency components can be scaled differently. For example, boosting low-frequency components can improve overall perceptual contrast and key tones. To achieve this, each frequency coefficient is multiplied by a scaling factor, which is increased for certain frequency bands to be emphasized, while other frequency bands, such as high noise-prone frequencies, may be reduced. Inspired by this concept, the conventional DCT approach is modified in both the forward and inverse transform parts in this study. Since it is impossible to preserve the original data without losing information about the spatial or temporal resolution of the signal and then upscaling, such operations usually result in some loss of quality, and loss of data accuracy.

In the proposed DRE-DCT method, the dynamic range is increased by emphasizing the low-frequency coefficients during the forward transform (similar to increasing perceived importance) and emphasizing or adjusting the high-frequency coefficients during the inverse transform. Since the low-frequency DCT coefficients mainly capture important features visible to the human eye, and the high-frequency coefficients capture more detailed features that may contain noise, there may be information loss in the forward DCT transform, which is usually not fully reversible. In order to achieve maximum compensation in the DCT inverse transformation, it is possible to try to faithfully reconstruct the amplified areas (low-frequency components) and minimize the introduction of perceptual artifacts. Thus, reducing the scope of this operation in terms of frequency manipulation leads to an increase in perceptual quality - a smooth emphasis on transitions in the frequency range, which minimizes abrupt changes and helps reduce quality loss.

The DRE-DCT method adds weighted frequency adjustments during both forward and inverse conversions, resulting in smoother transitions between conversion states and maintaining high image quality. This DRE-DCT (Dynamic Range Enhanced Discrete Cosine Transform) introduces new features by improving the traditional DCT equations for both onwards and reverse processes. The proposed DRE-DCT is an orthonormal transform with new contributions, such that the existing traditional DCT equations are improved in both onwards and reverse transforms, where 1D forward DRE-DCT and 1D inverse DRE-DCT are defined in Equations 4, 5 respectively under the special condition defined in Equation 6 as follows:


(4) $$\begin{array}{c} y\left(k\right)=\frac{N}{N+k}\sqrt{\frac{2}{N}}\alpha \left(k\right)\sum_{n=0}^{N-1}x\left(n\right)\cos\frac{\left(2n+1\right)k\pi }{2N}\\ k=0,\;1,\ldots N-1 \end{array}$$

(5) $$\begin{array}{c} x\left(n\right)=\sqrt{\frac{2}{N}}\sum_{k=0}^{N-1}\frac{N}{N-k}\alpha \left(k\right)y\left(k\right)\cos\frac{\left(2n+1\right)k\pi }{2N}\\ n=0,\;1,\ldots N-1 \end{array}$$

(6) $$\alpha (0)=\frac{1}{\sqrt{2}};\;\alpha (k)=1;\;k\neq 0.$$

This study uses the DRE-DCT technique to improve image quality, which is particularly useful for deep learning applications such as disease detection in bean leaves. High-quality images are important for accurate identification, as detailed features are important. The enhancement process starts by dividing the image into 16×16 subblocks. A forward DCT is then applied to each subblock using its pixel values, resulting in 256 DCT coefficients. These coefficients are divided into two types: one DC coefficient representing the low frequency component and 255 AC coefficients representing the high frequency components. DCT effectively compresses most of the image information into lower spatial frequencies, so the DC coefficient is critical because it carries more image data than the AC coefficients. The DC coefficient is prominent due to its low frequency and better visibility to the human eye, while the AC coefficients are associated with higher frequencies and are less visible.

Finally, DRE-DCT equations are applied to each block to improve image quality by increasing the dynamic range of the input blocks. Since the blocks are two-dimensional, 2D DRE-DCT is implemented by first running 1D forward DRE-DCT on each row, followed by each column, and then performing 1D inverse DRE-DCT in reverse order to return to the spatial domain with a higher dynamic range for each block.

**Figure 2:**
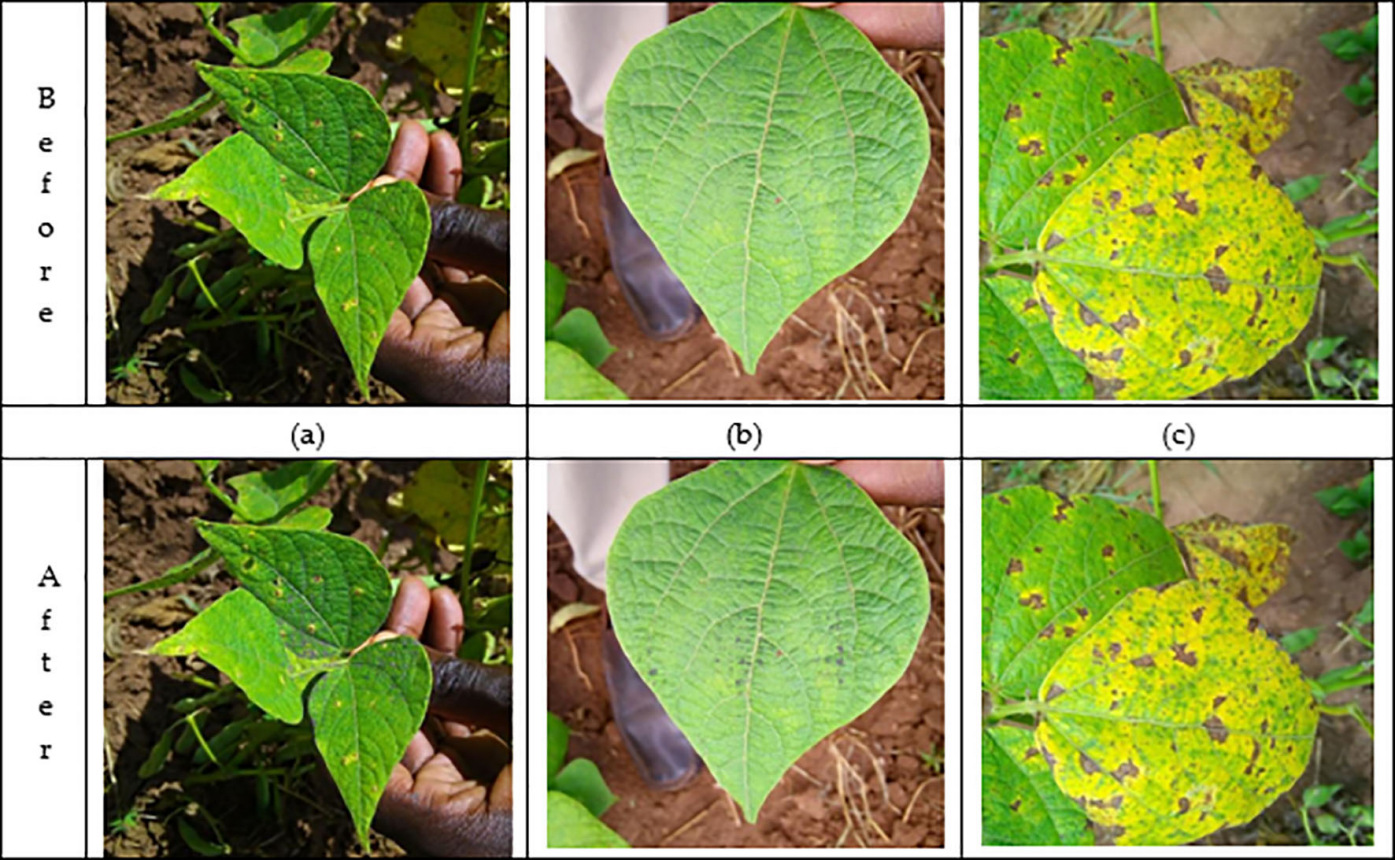
Before-after leaf images. Images **(a, c)** are standard views that may contain varying levels of disease, but lack clarity in highlighting subtle areas of disease extracted after enhancement. The difference between the before and after enhancement cannot be seen by the human eye. On the other hand, image **(b)** shows the effect of applying the DRE-DCT enhancement. DRE-DCT method extracts hidden high-frequency components in the original image, revealing additional disease areas. This magnification technique highlights complex textures and details, making it easier for experts to identify potential problem areas that are difficult to see in raw images. Additionally, it also facilitates deep learning methods to classify the bean leaves.

In **Figure 2**, three sample images, labeled (a), (b), and (c), show the effect of the DRE-DCT enhancement method on the visualization of diseased areas in bean leaf images.

### 2.2 Transformers

Transformers (Dosovitskiy, 2020) have been widely adopted for image classification due to their ability to capture complex relationships within data. To adapt the standard Transformer model for images, each input image $x\in R^{H\times W\times C}$ is split into smaller, non-overlapping patches image $X_{p}\in R^{N\times (P^{2}\in C)}$ , resulting in a sequence of flattened patches. Here, (*H*, *W*) are the image dimensions, *C* is the number of channels, and *(P, P)* defines the size of each patch. The total number of patches, *N*, is given by Equation 7:


(7) $$2N=\;\frac{HW}{P^{2}}$$

Each patch is then projected into a higher-dimensional embedding space using a linear transformation, as given in Equation 8:


(8) $$z_{i}=W_{i}x_{i}+b_{i}$$

where $W_{e}$ is the weight matrix and $b_{e}$ is the bias vector, ensuring that each patch is represented as a vector suitable for the attention mechanism.

The transformer encoder incorporates self-attention, which calculates the attention scores between patches to capture dependencies. Specifically, for each patch i, the attention with another patch j is determined using the dot product of their query and key vectors, normalized by the square root of the key dimension (see Equation 9):


(9) $$Attention_{i,j}=softmax\;(\frac{Q_{i}K_{j}^{T}}{\sqrt{D_{k}}})$$

This operation produces a contextually rich representation for each patch, which is further refined derivatively by a feed-forward network (FFN) applied independently to each patch embedding (see Equation 10):


(10) $$z_{i}^{\prime \prime \prime }=FFN\left(z_{i}^{\prime \prime }\right)$$

Additionally, positional encodings are added to retain the spatial structure of the input, enhancing the model’s ability to understand the arrangement of patches within the image. Finally, the output representation of the special [CLS] token, which aggregates information from all patches, is passed through a classification layer, yielding Equation 11:


(11) $$y=softmax(W_{c}z_{CLS}+b_{c})$$

This process generates logits that are converted into class probabilities, allowing the model to predict categories like “Angular Leaf Spot,” “Bean Rust,” and “Healthy.” The combination of patch embeddings, self-attention, and positional encoding enables the Transformer model to effectively learn and make predictions for complex visual tasks.

### 2.3 Transfer learning with transformers

Transfer learning requires adapting the pre-trained model to classify the bean leaf diseases. This study utilizes transfer learning with Google’s “*vit_base_patch16_224”* pre-trained model. The selection of this model is based on their immense training and performance in its prior challenges (Shehu et al., 2025c), as well as in similar plant disease detection use cases (Prashanthi et al., 2024; Singh et al., 2024) enhancing prediction accuracy while reducing computational time. The foundational Transformer architecture described previously serves as the backbone for these models, effectively processing images as sequences of patches.

The transfer learning process begins by loading the pre-trained “*vit_base_patch16_224”* transformer model and adjusting its architecture for the classification task. The classification algorithm encompasses preprocessing images, feature extraction from each model, and calculating class probabilities. The logits for classification are computed using the adapted output layer, as given in Equation 12:


(12) $$y=softmax\left(W_{c}^{\prime }z_{CLS}+b_{c}^{\prime }\right)$$

where $W_{c}^{\prime }$ and $b_{c}^{\prime }$ are the fine-tuned weight matrix and bias vector for the target task, respectively. Finally, the model undergoes fine-tuning on the iBean leaf diseases dataset (Singh et al., 2023), optimizing its parameters for accurate predictions.

## 3 Experimental work

A standard computer (MacBook M1 with 16GB of RAM, a 512 SSD and an integrated 8-core GPU) is used for experiments.

### 3.1 Datasets and experimental design

**Figure 3:**
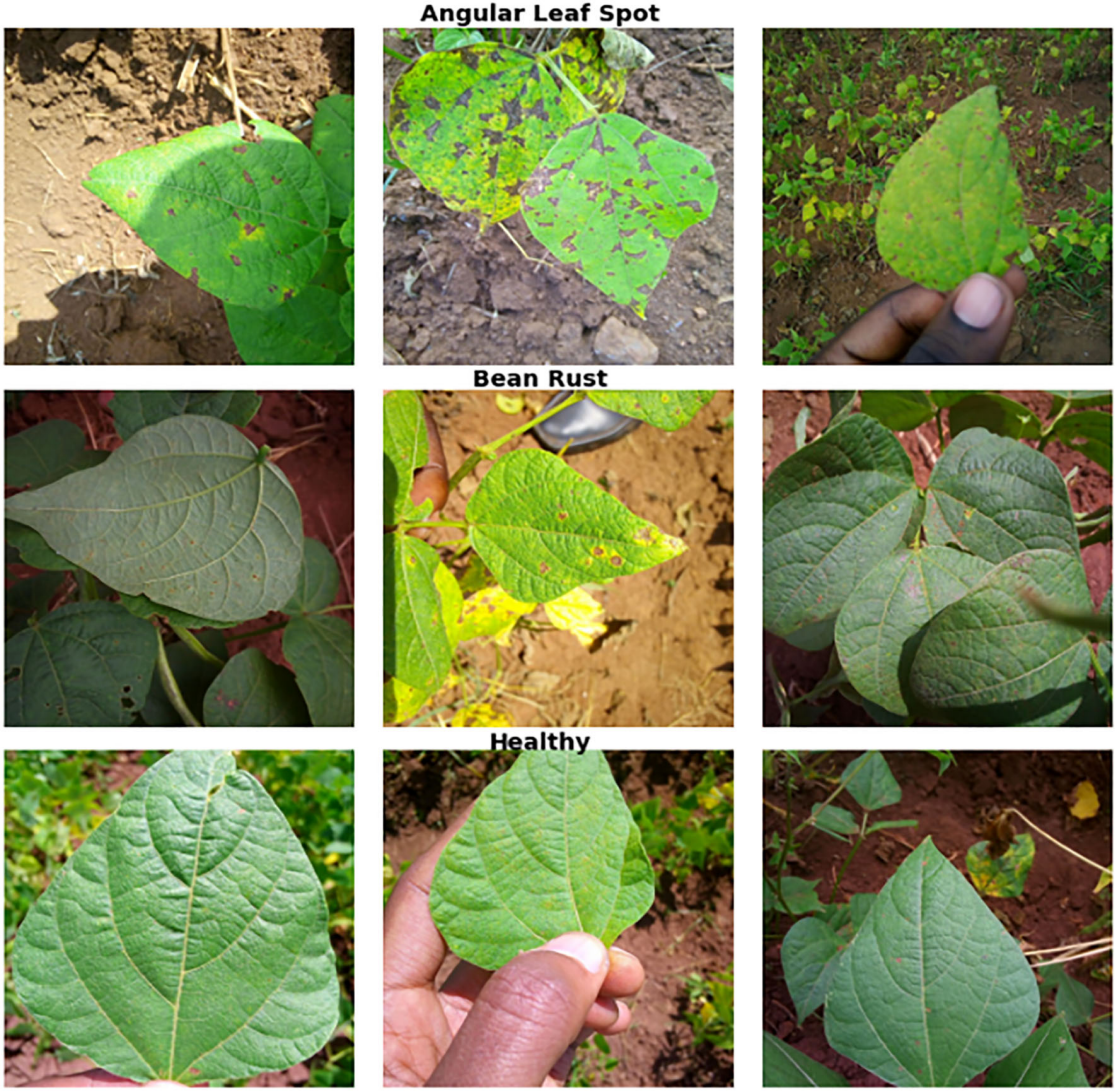
Sample bean leaves images from the iBean dataset.

This study utilized the iBean dataset (Makerere AI Lab, 2020) for analysis (see **Table 1**). The dataset consists of 1,295 images of bean plants, each carefully annotated to indicate various plant diseases and health conditions, including bean rust, angular leaf spot, and healthy leaves. This dataset was chosen because it is commonly used in many studies (Elfatimi et al., 2024; Noviyanto et al., 2024) making it well-suited for benchmarking our method against similar approaches. To facilitate effective analysis, we used 5-fold cross-validation, where each part of the dataset was tested. At each fold, 10% of the training set was used for validation, and all images were resized to a uniform dimension of 224×224 pixels. This standardization ensures consistency during model training and evaluation, allowing for meaningful comparisons of results across different sets. **Figure 3** illustrates sample images from the iBean dataset.

**Table 1:** Distribution of images in the iBean leaf dataset.

Class	Number of images
Healthy	428
Angular Leaf Spot	432
Bean Rust	436
Total	1,296

### 3.2 Data augmentation

Data augmentation was employed to enhance the diversity of the training dataset and improve the robustness of the model. The following transformations were applied to the images:

- Shift Left (0.1): This transformation shifts the image horizontally by 10 pixels, simulating slight lateral movements of the camera or object, helping the model learn spatial invariance.
- Rotate (15 degrees): The images were rotated by 15 degrees to introduce variability in orientation, allowing the model to recognize objects regardless of their rotational positions.
- Height Shift (0.1): This transformation shifts the image vertically by 10 pixels, mimicking minor changes in camera height, which aids in enhancing the model’s ability to handle positional variations.
- Horizontal Flip: The images were flipped horizontally, effectively doubling the dataset and teaching the model to handle mirrored versions of objects.
- Color Jitter (Brightness Adjustment 0.2): A brightness adjustment was applied to vary the lighting conditions within the images, making the model more robust to changes in illumination.

**Figure 4:**
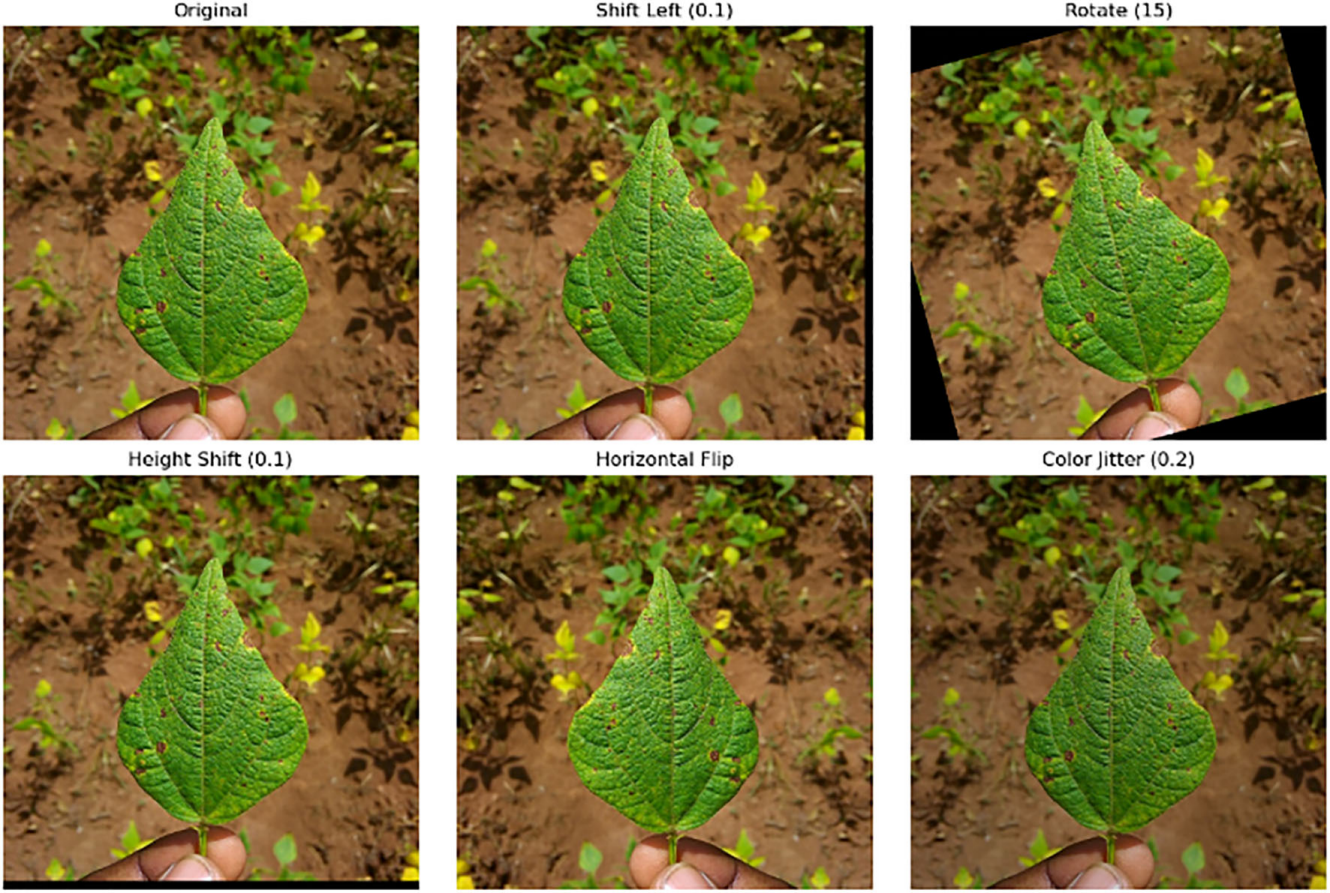
Sample images generated using the augmentation method.

These augmentations help in generating a more comprehensive dataset by simulating various real-world scenarios, thus reducing overfitting and improving the model’s generalization performance on unseen data. **Figure 4** presents sample images generated using the augmentation methods.

### 3.3 Parameter settings

A number of different parameters were tested during the training process, and the parameters listed in **Table 2** yielded the best accuracy results. Most of the other parameters were kept at their default settings.

**Table 2:** Optimized parameters giving the best accuracy result.

Parameters	Values
Image sizes	224 ×224
Learning rate	1×10^-4^
Optimizer	*Adam*
Batch sizes	16
Epochs	10

### 3.4 Ablation study

**Figure 5:**
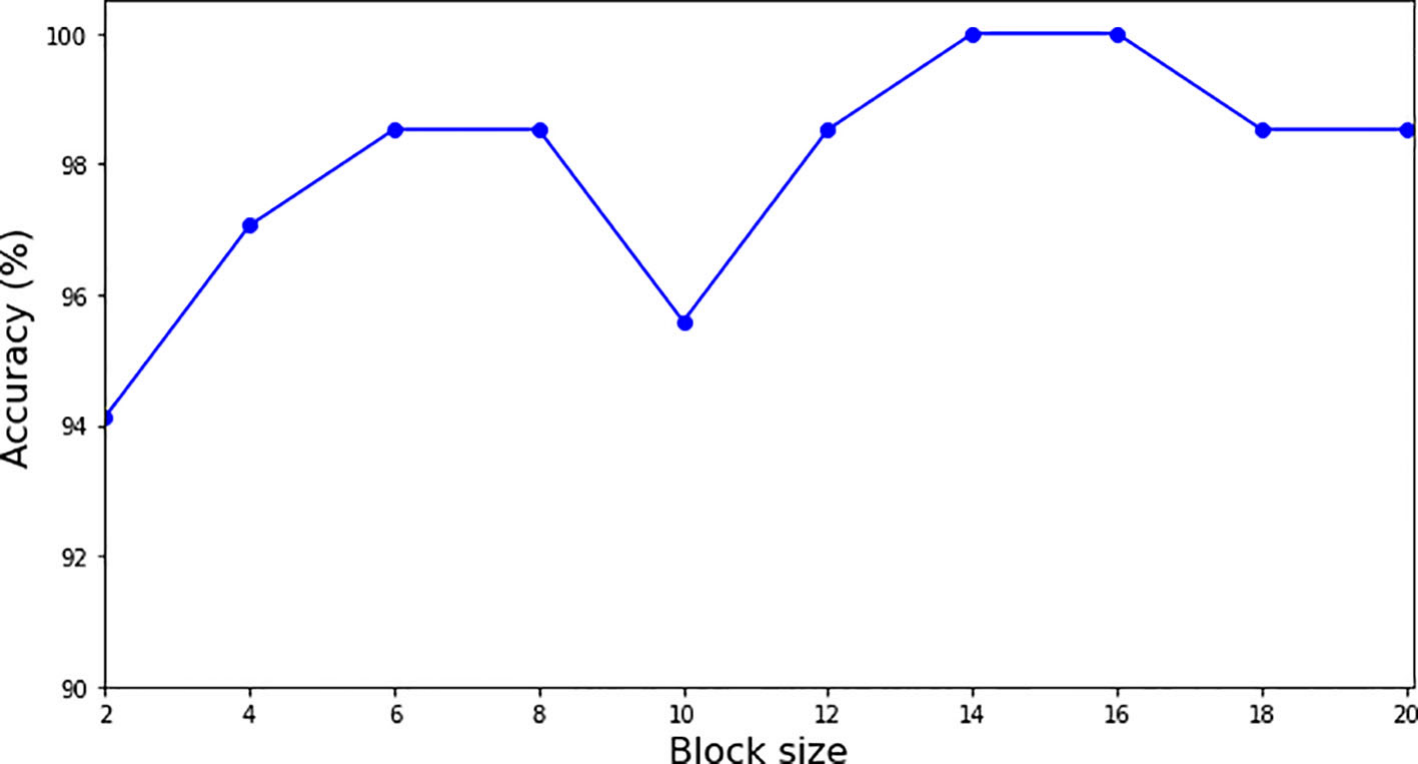
Graph for accuracy vs. block size. The model converges to 1.0 accuracy at the block size 14, 15 and 16.

We conducted an ablation study on a single fold using different block sizes to evaluate their impact on model accuracy. The results, illustrated in **Figure 5**, demonstrate that the model converges when the block size is set to 14, 15 and 16, achieving the highest accuracy of 100% on the tested fold. Based on these findings, we selected a block size of 16 for our analysis to ensure the best performance of the model.

As seen in **Figure 5**, the block sizes of 14, 15 and 16 are the saturation points for the growth of block size on the accuracy of classification. In the study, the analysis of classification accuracy in relation to block size revealed that block sizes of 14, 15 and 16 reached what is known as saturation points for accuracy. These saturation points indicate that these specific block sizes allow the model to achieve 100% classification accuracy, beyond which increasing the block size does not result in further improvements. This suggests an optimal balance between computational efficiency and feature extraction capability at these block sizes. At these saturation points, the model successfully leverages the enhanced features from the preprocessed images while maintaining rapid processing and minimal computational overhead. These findings have important implications for practical applications, offering guidelines for selecting block sizes that maximize both performance and efficiency when using the DRE-DCT for preprocessing in conjunction with Transformer Deep Learning models.

### 3.5 Evaluation of the proposed methods

In the “Method” column of **Table 3**, “DCT” refers to the results obtained using DCT. “Transformers” refers to the results obtained using vision transformers with transfer learning, specifically Google’s vit_base_patch16_224. “DCT-Transformers” refers to the results obtained when the two methods were combined.

**Table 3:** Results achieved by different methods on the iBean dataset.

Methods	Accuracy (%)
DCT	58.02
Transformers	95.92
DCT-Transformers	99.56

As shown in **Table 3**, the DCT method alone achieved an accuracy of 58.02%, while the vision transformers with transfer learning (Transformers) achieved a significantly higher accuracy of 95.92%. The combination of the two methods (DCT-Transformers) achieved the highest accuracy of 99.56% (with a precision of 0.9916, recall of 0.9912, and F1-score of 0.9912), outperforming each method when used independently.

These findings highlight the effectiveness of vision transformers with transfer learning as a robust approach to the bean leaf classification task, demonstrating superior performance compared to the DCT method alone. However, the combination of DCT and Transformers yielded the best results, suggesting that enhancing images with DCT before applying the transformer model enhances its feature extraction capabilities.

**Figure 6:**
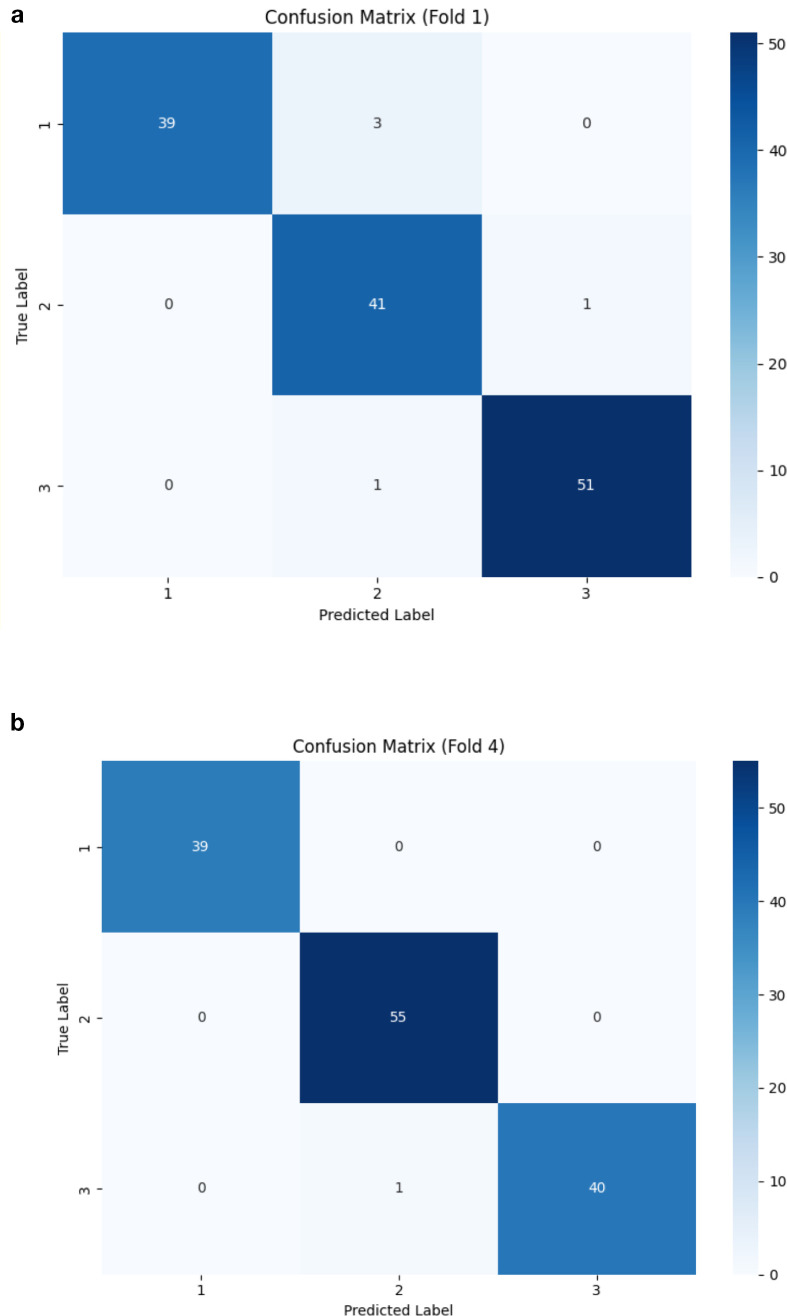
Confusion matrices of the worst performing folds, i.e. 1(see **a**) and 4 (see **b**). Note that 1 here represents healthy, 2 represents angular leaf spot, and 3 represents bean rust plant diseases.

**Figure 7:**
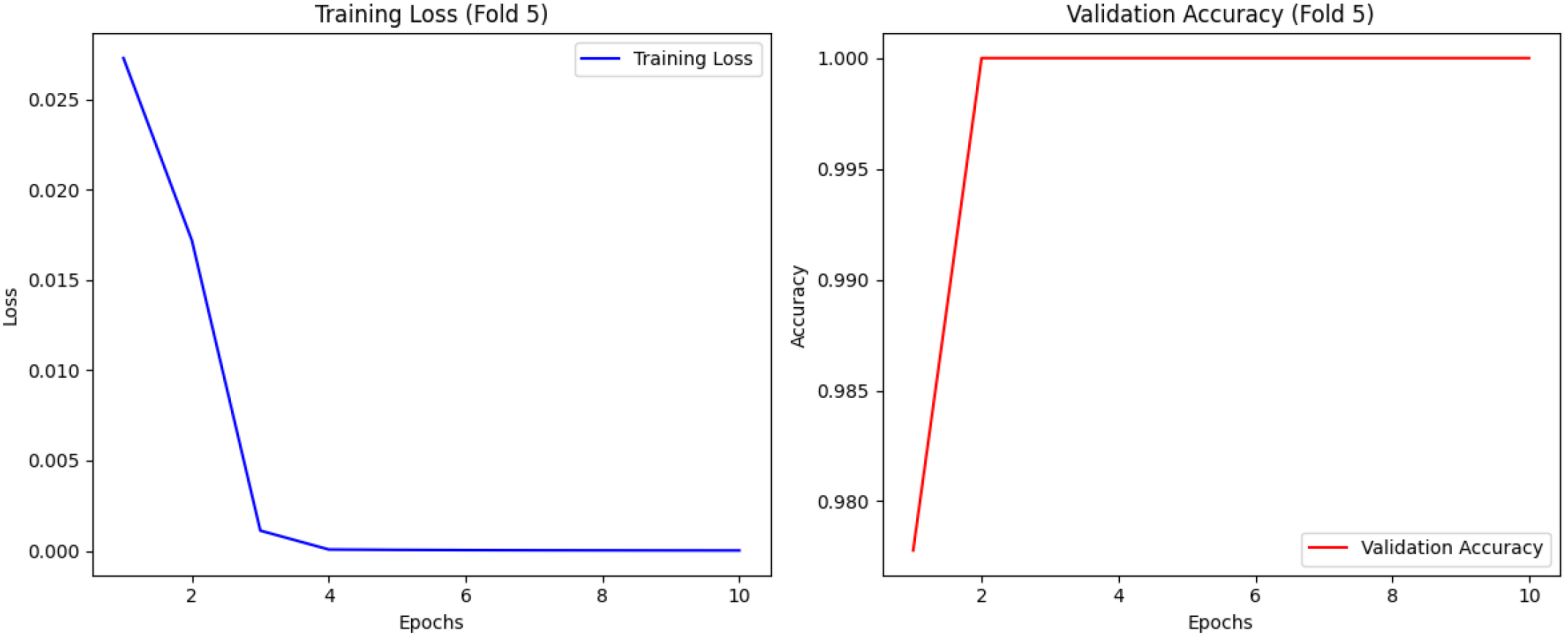
Training loss and validation accuracy graph for Fold 5.

**Figure 6** presents confusion matrices, highlighting the folds where the model performed the least effectively. While the proposed DCT-ViT model achieved 100% accuracy in three folds, it misclassified 5 images in the first fold and 1 image in the fourth fold. This indicates that, despite its high overall accuracy, the model exhibits inconsistencies in classifying certain classes across different folds. Specifically, the five misclassified images in fold 1 consist of 3 healthy, 1 angular leaf spot, and 1 bean rust image, while the single misclassified image in the fourth fold is a bean rust image. These findings highlight the need for further refinement to enhance the model’s robustness and generalization across all image categories, a critical factor for its effective application in real-world scenarios. Furthermore, the training loss and validation accuracy graph presented in **Figure 7** (see **Supplementary Figures 8**-**11** for the training loss/validation accuracy across all folds) provides evidence that the model did not overfit, as the validation accuracy closely follows the training trend without significant divergence.

Yet, the observed improvement in overall accuracy is noteworthy, as it suggests that the DCT method, which emphasizes important frequency components, complements the deep learning model’s ability to learn complex patterns. By incorporating DCT, the model is provided with enriched input data that enhances the overall performance of the transformer, making the combined approach more effective than using either method separately. This synergy highlights the importance of integrating traditional feature extraction techniques with advanced deep learning models to achieve superior performance, particularly in complex image classification, such as the bean leaf diseases classification.

### 3.6 Comparison with state-of-the-art methods

In the “Statistical Test” column of **Tables 4** and **5**, a “+” signifies that the proposed method significantly outperformed the compared method, whereas a “–” indicates instances where the compared method significantly outperformed the state-of-the-art method.

**Table 4:** Comparison of the proposed method with state-of-the-art deep networks on the iBean dataset.

DCT-Transformers	State-of-the-art methods	Statistical Test
99.56	GoogleNet - 91.36	+
	InceptionV3 - 93.37	+
	ShuffleNet - 91.82	+
	ResNet50 - 91.49	+
	MobilNetV3 - 92.19	+
	VGG19 - 93.73	+

**Table 5:** Comparison of the proposed method with similar studies.

DCT-Transformers	References	Statistical Test
99.56	(Elfatimi et al., 2024) El Fatimi et al. 92.97	+
	(Elfatimi et al., 2022) El Fatimi et al. 97.44	+
	(Singh et al., 2023) Singh et al. 91.74	+
	(Abed et al., 2021) Abed et al. 98.31	+
	(Noviyanto et al., 2024) Sunyoto et al. 96.90	+

**Table 4** compares the performance of the proposed DCT-Transformers method with state-of-the-art deep networks on the iBean dataset. As shown, the DCT-Transformers approach achieved an accuracy of 99.56%, outperforming all state-of-the-art methods, each of which achieved an accuracy of less than 94%.

Furthermore, a two-sample t-test confirmed that the differences in accuracy between the proposed DCT-Transformers method and each of the state-of-the-art deep networks were statistically significant (*p* <*.001*). This finding demonstrates the superiority of the combined approach over the state-of-the-art deep learning models on the iBean dataset.

### 3.7 Comparison with similar studies

**Table 5** compares the performance of the proposed DCT-Transformers method with results from similar studies. The DCT-Transformers approach achieved an accuracy of 99.56%, outperforming all referenced methods.

Furthermore, a two-sample t-test found that the differences in accuracy between the proposed DCT-Transformers method and each of the referenced studies were statistically significant (*p* <.*001*).

These results underscore the effectiveness of the combined DCT-Transformers approach, highlighting its ability to significantly outperform existing methods. The findings suggest that integrating DCT with vision transformers enhances the model’s performance by leveraging DCT’s frequency-based feature extraction alongside the advanced pattern recognition capabilities of the transformer model. This combined approach not only delivers superior accuracy but also demonstrates the potential of hybrid methods to advance the state-of-the-art in image classification, particularly in bean leaf classification tasks.

## 4 Discussion and future study

The proposed DRE-DCT method employs the advanced compression capabilities of the DCT function, which is specifically designed to increase the dynamic range of images. This strategic enhancement makes it easier to extract complex details and high-frequency components which are the elements often imperceptible to the human eye while preserving the integrity and quality of the original images. By focusing on this high-frequency detail, our approach provides essential data for accurate image analysis, which is particularly useful in agricultural applications where detailed visibility of leaf texture and disease patterns is important.

Although Haar Wavelet Transform (HWT) (Bulut, 2022) and Fourier Transform (FT) can be used to improve dynamic range, its compression efficiency is significantly lower compared to DCT. This limitation is important because compression not only affects storage and transmission efficiency, but also affects the ability of algorithms to target relevant image features. Our method exploits the excellent capabilities of DRE-DCT to selectively preserve and enhance high-frequency components without compromising the benefits of compression, which has been shown to be essential for detailed extraction of diseased bean leaves.

Standard JPEG compression typically uses a default block size of 8×8 pixels to balance quality and efficiency. However, we employed 16×16 block size as the optimum parameter for the DCT block size since the Transformer deep learning model employs 16×16 patch size in transfer learning. This modification facilitates the capture of more complex image features critical for bean leaf disease detection and underpins our exceptional achievement in detecting bean leaf diseases with unprecedented classification accuracy, laying the foundation for advances in precision agriculture diagnostics.

The exceptional performance and 99.56% classification accuracy of Transformers when paired with DRE-DCT processed input images can be attributed to several factors. Firstly, DRE-DCT pre-processes images, increasing their dynamic range and emphasizing high-frequency components. This ensures that even fine details are highlighted, which are important for distinguishing between healthy and diseased leaves. When presented with feature-enriched data, the Transformer’s attention mechanisms can more efficiently focus on relevant parts of the image, resulting in better classification. On the other hand, DRE-DCT reduces noise while preserving important high-frequency information. This balance helps the Transformer model by providing cleaner and more distinct input features, minimizing the risk of misclassification due to noise artifacts. Additionally, by increasing dynamic range, DRE-DCT makes fundamental differences in leaf texture and color more apparent. Transformers excel at capturing these variations due to their ability to handle complex and high-dimensional data representations, which increases accuracy. Besides, DRE-DCT improvements result in images that preserve key pattern details and contrasts critical for deep feature learning. Transformers use this rich data representation to learn distinctive features that are key to accurate classification. In essence, Transformers, with their multi-head attention mechanisms, can simultaneously monitor information from different performance areas in different frequency domains. Moreover, DRE-DCT’s high-frequency emphasis helps transformers use these attentional mechanisms to identify key indicators of disease that might otherwise be overlooked. Finally, using a block size of 16×16 in DRE-DCT preprocessing aligns well with the transformer architecture that employs the same patch size, yielding 100% accuracy on a single fold and 99.56% accuracy across all five folds, highlighting the main contribution of this study.

Using transformers with transfer learning, specifically the Google ‘*vit_base_patch16_224’* model, and the proposed method achieved an impressive accuracy of 95.92%. However, with the inclusion of DRE-DCT as a pre-processing step before feeding the images to the model, the accuracy remarkably increased to 99.56% across all folds. The significant improvement highlights the effectiveness of DRE-DCT in enhancing model performance. DRE-DCT helps by reducing image noise and highlighting critical frequency components, which improves feature extraction by the model. This pre-processing technique effectively emphasizes essential patterns in the images while minimizing irrelevant variations, leading to more accurate and robust predictions.

Overall, the amalgamation of DRE-DCT’s preprocessing capabilities and the Transformer’s sophisticated modeling capacity provides a robust framework that leverages both enhanced feature availability and advanced learning mechanisms, resulting in exceptional classification accuracy in comparison to methods utilizing only Transformers for classification (Sun et al., 2025).

The current study evaluated the performance of the proposed method only on bean leaf disease images. To effectively investigate its performance on a broader range of plant diseases, it is necessary to test the method on diverse crop datasets under varying conditions (Shehu et al., 2025a). Therefore, future research should explore its effectiveness on other plant leaf diseases, such as those affecting tomato (Shehu et al., 2025b), corn (Sebastian et al., 2024), rice (Borhani et al., 2022), cassava (Thai et al., 2023), and others.

Additionally, we recommend the development of an adaptive, automated block size assignment mechanism. Such a system would dynamically adjust block sizes based on the specific characteristics of each input image, thus enabling a parameterless optimization technique. This mechanism would enhance adaptability and performance, allowing the DRE-DCT method to tailor its feature enhancement capabilities optimally across differing image scenarios.

## 5 Conclusions

In conclusion, this study presents a novel approach for the early detection of bean leaf diseases using a Transformer deep learning model enhanced by dynamic range enhanced discrete cosine transform (DRE-DCT) preprocessing, referred to as DCT-Transformers. By modifying the input images to highlight imperceptible details and high-frequency components, our method achieved perfect classification accuracy on the iBean dataset. The effectiveness of DRE-DCT is demonstrated by a significant improvement in feature extraction, enabling the Transformer model to classify diseased leaves with exceptional precision. The study further shows that classification accuracy peaks at 100% (on a single fold) when a DCT block size of 16×16 is paired with the same 16×16 patch size in the Transformer model. Our experiments on the iBean leaf disease dataset yielded 99.56% (across all folds) classification accuracy, underscoring the reliability and robustness of the approach. The results confirm the excellent capacity of the DRE-DCT method to enhance image preprocessing and its significant potential for improving the detection of agricultural diseases. Overall, this research not only advances the field of plant pathology but also provides a critical tool for sustainable agricultural practices, with implications for broader applications across various plant species and diseases.

## Data Availability

The datasets utilized in this study can be found in online repositories. The name of the repository(ies) and access number(s) can be found below: The Makere iBean dataset can be downloaded from the following link: https://github.com/AI-Lab-Makerere/ibean/. The code implemented in this study can be accessed via: https://github.com/harisushehu/bean-leaf-diseases-detection.

## Supplementary material

Supplementary Figure 8 Training loss and validation accuracy graph for Fold 1.

Supplementary Figure 9 Training loss and validation accuracy graph for Fold 2.

Supplementary Figure 10 Training loss and validation accuracy graph for Fold 3.

Supplementary Figure 11 Training loss and validation accuracy graph for Fold 4.
