# Supplementary material for: A novel transformer using dynamic range-enhanced discrete cosine transform for detecting bean leaf diseases
Source: Front Plant Sci. 2025 Aug 29;16:1624373. doi: 10.3389/fpls.2025.1624373 (PMC12426951; doi:10.3389/fpls.2025.1624373)
Supplement: Supplementary Figure 8 — Training loss and validation accuracy graph for Fold 1. [file DataSheet1.docx]

**Supplementary Materials**

**
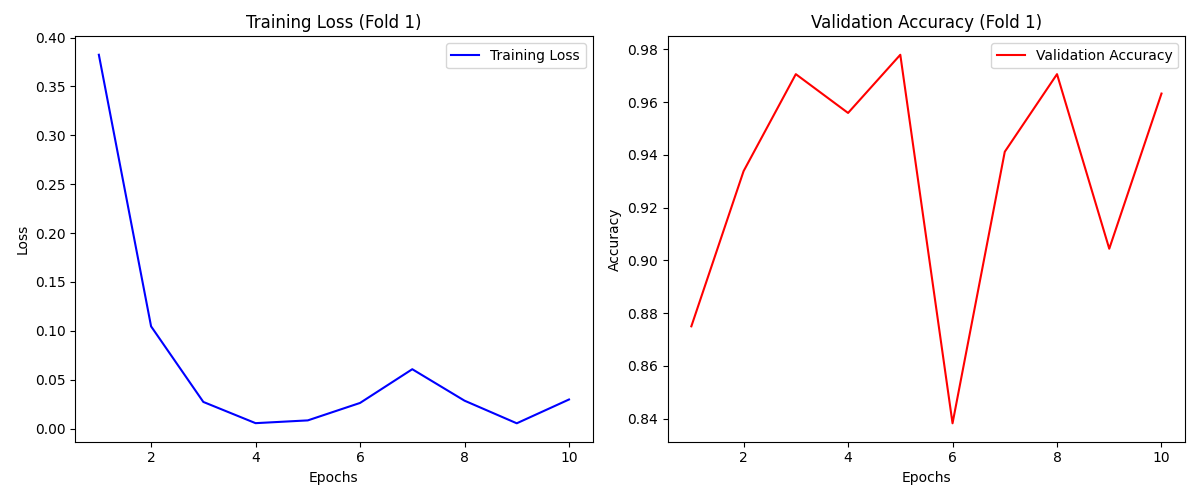
**

**Figure 8: Training loss and validation accuracy graph for Fold 1.**

**
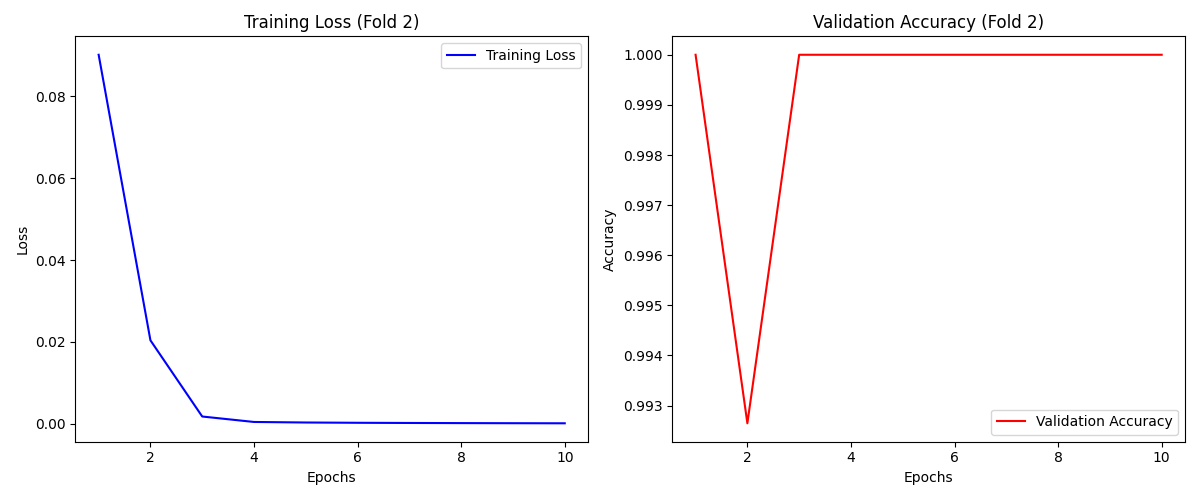
**

Figure 9: Training loss and validation accuracy graph for Fold 2.

**
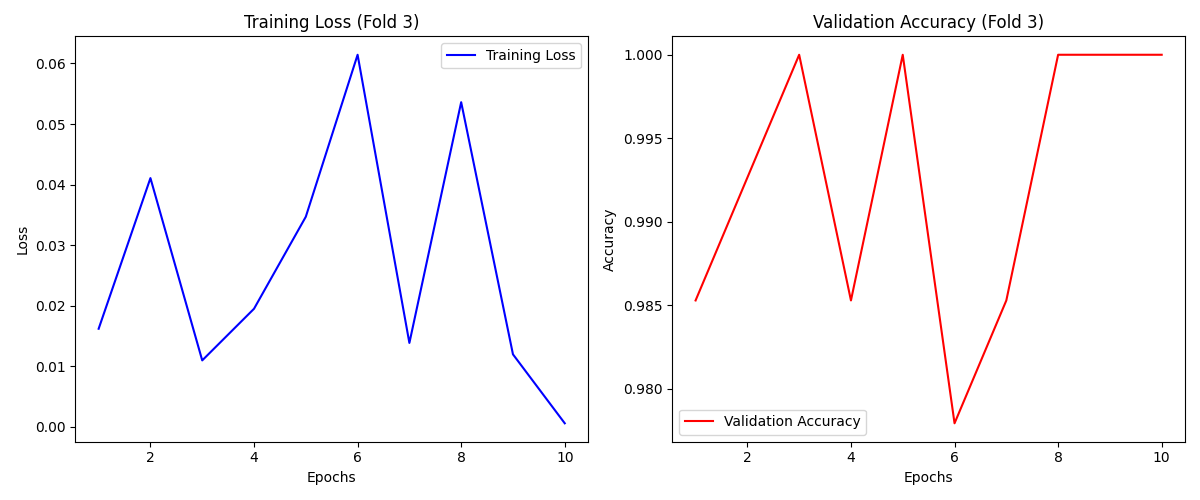
**

Figure 10: Training loss and validation accuracy graph for Fold 3.

**
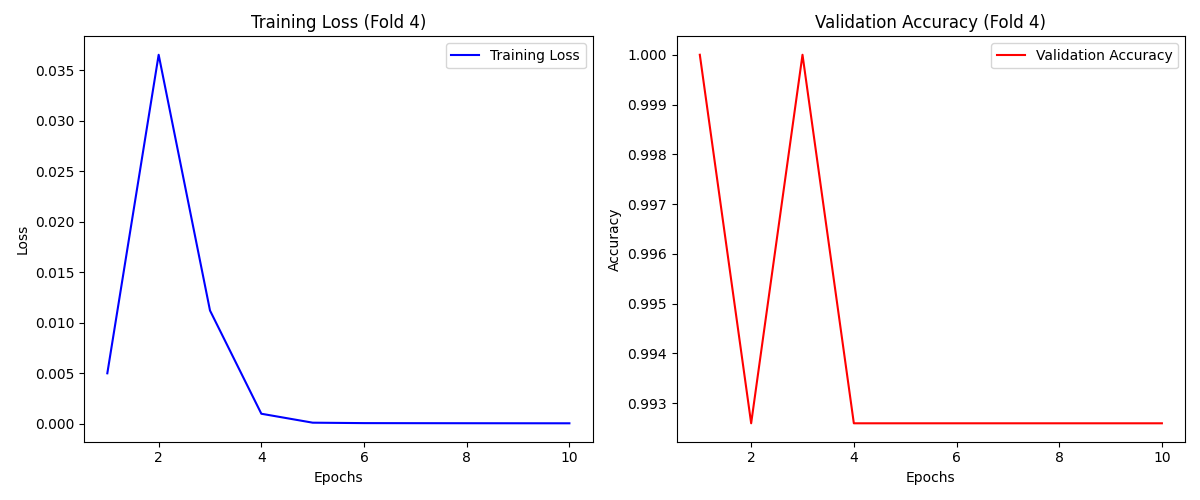
**

Figure 11: Training loss and validation accuracy graph for Fold 4.
